# Mulberry Leaf Protein: Extraction Technologies, Functional Attributes and Food Applications

**DOI:** 10.3390/foods14152602

**Published:** 2025-07-24

**Authors:** Rongxiang Xue, Lichao Chen, Chao Sun, Abrar Muhammad, Yongqi Shao

**Affiliations:** 1 Institute of Sericulture and Apiculture, College of Animal Sciences, Zhejiang University, Hangzhou 310058, China; 3220104767@zju.edu.cn (R.X.); 3210103113@zju.edu.cn (L.C.); abrar_ento334@zju.edu.cn (A.M.); 2 Analysis Center of Agrobiology and Environmental Sciences, Zhejiang University, Hangzhou 310058, China; chaosun@zju.edu.cn; 3 Key Laboratory of Silkworm and Bee Resource Utilization and Innovation of Zhejiang Province, Hangzhou 310058, China; 4 Key Laboratory for Molecular Animal Nutrition, Ministry of Education, Hangzhou 310058, China

**Keywords:** mulberry leaf, mulberry leaf protein, preparation process, nutrition, bioactivity

## Abstract

In the context of a rapidly expanding global population, mulberry leaf protein emerges as an emerging source of plant protein, with most applications currently at Technology Readiness Level (TRL), presenting substantial potential for application in functional foods and nutraceuticals. This paper analyzes three key advantages of mulberry leaf protein. Firstly, the abundant and inexpensive production of mulberry leaves establishes a solid foundation for large-scale protein extraction. Secondly, advancements in the preparation processes and production technology for mulberry leaf protein have further enhanced its viability. Thirdly, mulberry leaf protein boasts excellent nutritional value and outstanding functional properties, along with multiple biological activities, including antioxidant effects, aging delay, and blood-pressure-lowering activity. These superior qualities considerably broaden its range of applications. Furthermore, this paper evaluates existing research (before 30 June 2025) while exploring prospective avenues for future investigation. The findings of this review are important for enhancing the understanding of the potential applications of mulberry leaf protein in food science and nutrition. The aim is to provide new ideas for the efficient utilization of mulberry leaf protein resources and the establishment of a global food security system.

## 1. Introduction

**Figure 1:**
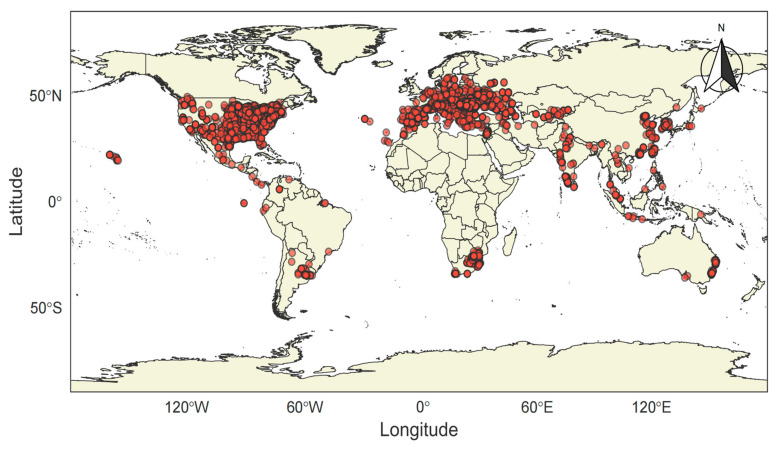
Geographical distribution of *Morus alba* L. (Data from GBIF.org (12 July 2025) GBIF Occurrence. Download https://doi.org/10.15468/dl.daws82 (assessed on 12 July 2025). Figure was created by using RStudio 2024.09.1+394 “Cranberry Hibiscus” Release).

Mulberry (*Morus alba* L.) is a woody, perennial plant belonging to the mulberry family (Moraceae). It is both flowering and deciduous, with a wide distribution across Asia, Europe, and America (Figure 1) [1,2,3]. The cultivation of mulberry in China dates back to ancient times [4]. During this period, mulberry leaves were used not only for rearing silkworms but also as a traditional Chinese herb, known for their documented efficacy in treating conditions related to wind–heat accumulation, lung issues, dryness, liver ailments, and visual impairment [5].

In recent years, rapid advancements in biotechnology have drastically increased public knowledge about mulberry leaves. From a chemical composition perspective, mulberry leaves contain nutrients such as crude protein (27.6–37.4%), soluble carbohydrates (5.87–15.0%), and crude fiber (9.90–13.9%). Additionally, they are rich in various bioactive compounds, including 1-deoxynojirimycin, phenolic acids, flavonoids, alkaloids, and γ-aminobutyric acid (GABA) [6,7,8]. As a result, mulberry leaves are regarded as a food resource with considerable development potential and are currently used to produce a variety of food products, such as bread, biscuits, pastries, and beverages [9,10].

With the continuous growth of the global population, providing sufficient protein for everyone in an environmentally sustainable manner has become an important issue that urgently needs to be addressed [11]. Fortunately, plant-based proteins present a practical solution to this nutritional challenge due to their cost-effectiveness and widespread availability in many regions worldwide [12,13]. Mulberry has a long history of cultivation in Asia, particularly in China, where it is traditionally associated with sericulture and recognized in traditional Chinese medicine for its therapeutic properties [4]. For centuries, its leaves have been utilized to feed silkworms and to create herbal remedies aimed at addressing metabolic disorders and inflammation. These longstanding uses have paved the way for contemporary research into the broader bioactive and nutritional potential of mulberry. Today, advancements in extraction and processing technologies have expanded the applications of mulberry leaves, establishing them as a promising source of functional plant protein.

**Figure 2:**
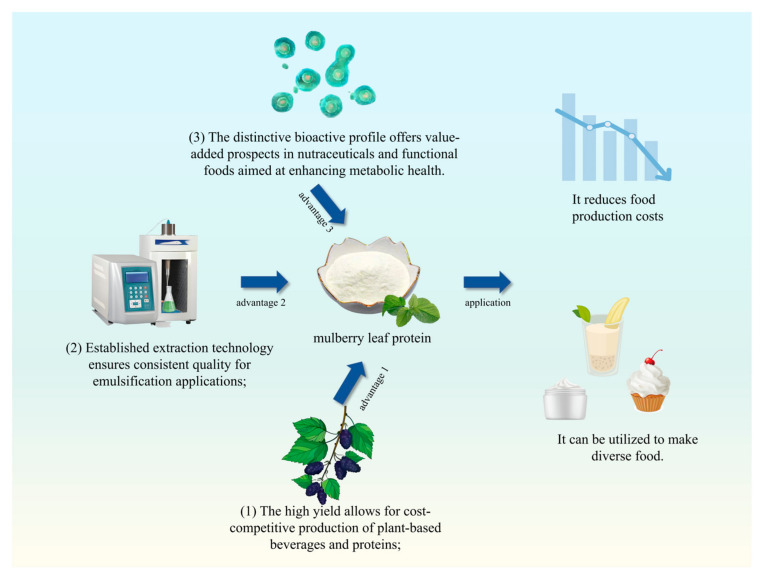
The three major development advantages of mulberry leaf protein.

**Figure 3:**
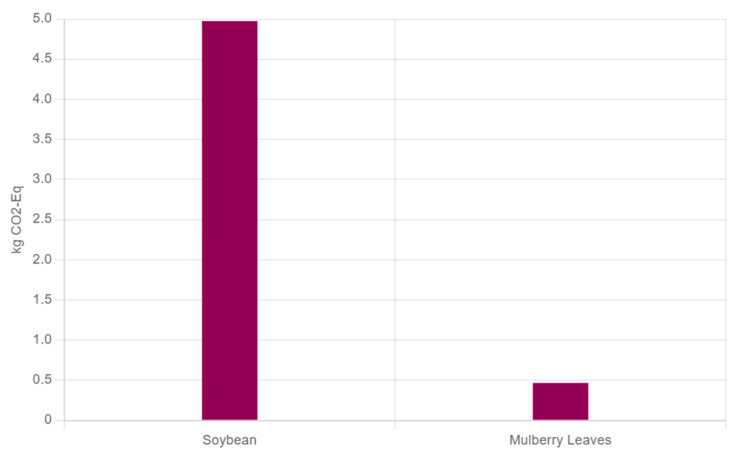
The impact of producing 1 kg of soybeans and 1 kg of mulberry leaves on climate change.

Mulberry leaf protein (MLP), one of the vital plant protein resources, shows tremendous potential for development (Figure 2). The three key advantages illustrated in Figure 2 present clear commercial opportunities: (1) the high yield allows for cost-competitive production of plant-based beverages and proteins; (2) established extraction technology ensures consistent quality for emulsification applications; and (3) the distinctive bioactive profile offers value-added prospects in nutraceuticals and functional foods aimed at enhancing metabolic health. In China, the substantial yield and low cost of mulberry leaves provide a solid foundation for large-scale production [14,15]. In recent years, numerous researchers have optimized the preparation process of MLP, and the yield of MLP has reached 16.29% through microbial fermentation. The technology for MLP production, such as the ultrasound/microwave-assisted extraction method, has become increasingly mature, offering technical support for large-scale production [16]. MLP possesses high nutritional value (it is rich in 17 kinds of amino acids, with a balanced proportion of amino acids) and excellent functional properties, along with bioactive effects such as antioxidant (IC_50_ value is 87 µg/mL), anti-aging, and blood pressure-lowering activities. These characteristics collectively enhance their application prospects in functional foods and nutraceuticals [17]. Compared to widely used plant proteins such as soy, pea, and rice, MLP offers a moderate protein content with a balanced amino acid profile. Moreover, MLP production is potentially more environmentally sustainable than that of soybean protein, offering additional ecological advantages. Data from the ecoinvent database (https://ecoinvent.org, assessed on 17 July 2025) analyzed by OpenLCA (https://www.openlca.org, assessed on 17 July 2025) shows that producing 1 kg of fresh mulberry leaves emits 0.466 kg of carbon dioxide, while producing 1 kg of fresh soybeans releases 4.975 kg (Figure 3). With mulberry leaves containing approximately 8% protein and soybeans about 40%, they produce less CO_2_ when providing equivalent protein. By supplementing traditional protein sources with mulberry leaves, this approach not only eases supply pressures on conventional protein sources but also contributes to reducing overall carbon emissions.

This review synthesizes current literature on MLP, critically examining its preparation processes, nutritional profile, functional properties, and bioactivities. It also explores potential future research directions. The imperative to develop sustainable, nutritious, and functional protein sources has never been greater. MLP emerges not merely as an alternative protein but as a uniquely positioned resource combining high-quality protein with inherent bioactive compounds. Successfully harnessing MLP at scale could therefore contribute to a more resilient and health-promoting global food system, moving beyond basic protein sufficiency towards enhanced nutritional quality and functionality. The findings of this review provide important insights into the possible applications of MLP in food science and nutrition, offering novel perspectives on the efficient utilization of MLP resources and the establishment of a global food security system.

## 2. Literature Search Strategy

An electronic literature search was conducted using CNKI (China National Knowledge Infrastructure), Web of Science, Bing Scholar, and PubMed until June 2025. Additional articles were identified and obtained from references in the retrieved articles. Search terms included combinations of the following: mulberry leaf protein, mulberry, anti-oxidative, functional properties, etc. For the purpose of this review, the search was restricted to experimental, in vitro, in vivo, and clinical studies published in Chinese and English that address the phytochemical components, functional properties, and biological activities (especially anti-oxidative effects) of mulberry leaf protein and related mulberry-derived materials.

## 3. Extraction Technologies for MLP

This section reviews MLP extraction methodologies, comparing their mechanistic bases, efficiency, and impacts on final product quality (Table 1).

### 3.1. Alkali-Acid Precipitation Method and Salting-Out Method

Proteins undergo denaturation and aggregation, resulting in precipitation, at isoelectric points or in strongly acidic or alkaline environments [42]. Utilizing this property, Wang et al. [18] extracted MLP from fresh leaves using water as the extraction solvent. They induced protein flocculation at 75 °C and precipitation at pH values of 5.0, 8.0, and 13.0, ultimately achieving an MLP yield of 5.17%. Liu et al. [43] used young mulberry stems and leaves as raw materials, extracting at 40 °C with 0.5% NaOH for 25 min, resulting in a maximum MLP extraction rate of 2.73%. Similarly, Zhang et al. [44] reached comparable conclusions. While this method is simple and cost-effective, the MLP obtained is prone to denaturation, leading to relatively low yield and purity [19,20].

The salting-out method extracts proteins by utilizing the varying precipitation thresholds of different substances in a salt solution [45]. Yang et al. [21] utilized a Na_2_HPO_4_-citrate buffer solution to extract MLP, suppressing protease activity in mulberry leaves by incorporating EDTA-2Na to minimize MLP degradation. They established the optimal concentration of EDTA-2Na by creating four concentration gradients at 0, 3, 5, and 7 mmol/L. Through comparison of protein degradation curves at various EDTA-2Na concentrations, they determined that the inhibition was most effective at five mmol/L. Their method preserves protein bioactivity without compromising it and better maintains the nutritional value of MLP; however, the yield is relatively low at just 1.38%. Moreover, although EDTA-2Na is filtered out during ultrafiltration with minimal residual amounts and does not directly influence the determination of protein yield by the precipitation method, incomplete elimination of EDTA-2Na could potentially affect subsequent protein functional studies due to its metal-chelating properties.

Zhang et al. [46] conducted a thorough comparative analysis of the effects of various protein precipitation methods and drying techniques applied to mulberry leaves on the amino acid content, composition, and nutritional value of MLP. The study found that different protein precipitation methods influence the amino acid content and composition of MLP, while the protein drying methods did not affect these parameters. Specifically, MLP extracted through acid precipitation exhibited higher levels of various amino acids and total amino acids (TAAs) compared to samples prepared using the salting-out method. Nonetheless, the MLP derived from both methods showed relatively similar characteristics in terms of the ratio of essential amino acids (EAAs) to total amino acids (TAA) and non-essential amino acids (NEAA).

Sun et al. [47] further explored the effects of various extraction methods, including heat treatment, acid precipitation, acid-heat treatment, and salting-out treatment, on the functional properties of MLP. Their study revealed that different precipitation methods significantly impact the functional properties of MLP (*p* < 0.05), with MLP prepared via the salting-out method demonstrating optimal performance in terms of solubility, foaming capacity, emulsifying activity, and oil absorption capacity. Electron microscopy observations showed that MLP prepared by the salting-out method displayed a typical honeycomb-like structure, which provided excellent physical entrapment effects, resulting in superior water-holding capacity and oil absorption properties.

In addressing whether different drying methods affect the functional properties of MLP, Liang et al. [48] conducted an in-depth investigation. The study found that hot air drying of mulberry leaves significantly enhances the digestive efficiency and solubility of MLP (*p* < 0.05); however, this process also leads to a degree of protein degradation or structural denaturation. In contrast, vacuum freeze-drying of mulberry leaves causes minimal disruption to the MLP structure, effectively preserving and protecting the unique properties of the protein.

### 3.2. Microbial Fermentation

Certain microorganisms can produce substances capable of precipitating MLP, such as lactic acid produced by *Lactobacillus* [49]. Therefore, microbial fermentation can be employed for MLP extraction. This method does not require heating and generates no waste or pollution. However, the fermentation process is time-consuming and may result in nutrient loss. Additionally, large-scale production requires a complete set of fermentation equipment, resulting in higher production costs [22]. Qu et al. [23] attempted to use *Bacillus subtilis* to ferment dried mulberry leaves for MLP production in an Erlenmeyer flask (250 mL), achieving a yield of 16.29%. In vitro digestion experiments confirmed that the digestibility of MLP prepared by this method was significantly improved (*p* < 0.05), with its digestion rate increasing by 4.29 percentage points compared to before fermentation. Fan et al. [50] employed the response surface methodology to precisely regulate fermentation conditions in an Erlenmeyer flask (100 mL) and identified the optimal parameter combination: a sucrose concentration of 6%, an ammonium sulfate ratio of 1.5%, and a fermentation temperature of 30 °C. Under these conditions, the final yield of MLP increased to 246.8 mg/g, achieving a substantial 1.57-fold enhancement compared to the unoptimized yield of 157.5 mg/g.

### 3.3. Foam Separation Method

Foam separation technology is a method that relies on the surface adsorption mechanism of interfacial chemistry, utilizing foam as the separation medium [51]. By introducing gas into the solution to generate foam, it promotes the expansion of foam through the strong adsorption of surface-active components (such as proteins) at the gas–liquid interface. Subsequently, during the defoaming step, effective separation or enrichment of surface-active substances from the liquid phase is achieved. Also known as foam adsorption separation technology, it has demonstrated exceptional efficacy in protein purification and separation, offering several advantages such as simple equipment, straightforward operation, low energy consumption, environmental friendliness, and high efficiency. Its application scope is extensive, suitable not only for the separation of microalgae and natural organic products but also for the effective extraction of various substances, including minerals [24]. Research indicates that by adjusting operational factors such as airflow rate, pH value, temperature, and tilt angle, performance metrics, including enrichment ratio and recovery efficiency, can be improved, leading to more efficient and precise separation outcomes [25]. Liu et al. [26] successfully employed foam fractionation technology to prepare MLP and utilized response surface methodology to define the optimal process parameters: diluting the sample to one-fortieth of its original concentration, adjusting the pH to 5.5, setting the ionic strength at approximately 0.18 mol/L, and maintaining the temperature at 25 °C. Under these optimized conditions, the actual recovery rate of MLP reached as high as 92.50%, and the enrichment ratio increased to 7.63. According to the Coomassie Brilliant Blue colorimetric method, the initial MLP content in the solution was determined to be 16.8%, and the calculated yield of MLP extracted by foam separation was 15.5%. This achievement strongly validates the efficacy of foam fractionation technology in MLP preparation. However, when tasked with separating high-concentration solutions, the efficiency of this technology appears to be relatively low, revealing certain limitations within its application scope [27].

### 3.4. Cellulase-Assisted Extraction Method

Cellulase, as an enzyme capable of catalyzing the decomposition of cellulose, has been proven to be highly effective in breaking down cellulose within mulberry leaf cell walls and intercellular substances [52]. Specifically, cellulase is a complex of enzymes, including endoglucanases, exoglucanases, and β-glucosidases, which act synergistically to hydrolyze cellulose into glucose units. The core of the catalytic process lies in cleaving the 1,4-β-D-glycosidic bonds in cellulose molecules, a step that is crucial for polysaccharide degradation. This process disrupts the compact structure of the cell wall and reduces mass transfer resistance, thereby promoting the release of MLP and improving extraction efficiency [53]. Zhu et al. [28] selected mulberry leaves as the raw material and employed a cellulase-assisted extraction method to obtain MLP. Through meticulous analysis using response surface methodology, they successfully determined the optimal process parameters for extracting proteins from mulberry leaves with cellulase: enzyme dosage of 4%, extraction time of 2 h, and solid-to-liquid ratio of 1:38 (g/mL). Under these conditions, the protein content in the mulberry leaf sample was measured at 49.056 mg, with a yield of 0.98%. Although this extraction technique exhibits high selectivity and mild reaction conditions, its drawbacks include relatively slow extraction rates and the tendency to introduce enzyme protein impurities during operation, which affects purity [28,29]. To address these challenges, strategies such as enzyme immobilization, purification steps post-extraction, or the use of purified enzyme preparations can be employed to reduce contamination and improve product purity.

### 3.5. Ultrasound/Microwave-Assisted Extraction Method

Ultrasound/microwave can disrupt the cell wall structure and increase cell wall permeability, which is precisely the principle utilized by ultrasound/microwave-assisted extraction methods [54,55]. Yang et al. [30] revealed the optimal conditions for ultrasound-assisted salting-out extraction of MLP in their study: liquid-to-solid ratio of 43:1 (mL/g), 390 W ultrasonic treatment for 20 min, extraction time of 40 min, and NaCl concentration of 0.42%. Under these conditions, the extraction yield of mulberry leaf protein reached 9.19%. Wang et al. [31] also reached similar conclusions in their study. Zhou et al. [32] employed ultrasound-assisted alkali dissolution and acid precipitation to extract MLP, determining the optimal process parameters for mulberry leaf protein extraction as follows: solid-to-liquid ratio of 1:24 (g/mL), extraction temperature of 30 °C, ultrasonication time of 4.9 min, ultrasonication power of 235 W, extraction solution pH of 11, and extraction time of 60 min. Under these conditions, the extraction yield of mulberry leaf protein increased to 34.12%. This result coincides with the experiments conducted by Yin et al. [33] and Yang et al. [34]. Wu et al. [35] prepared mulberry leaf protein through ultrasonic extraction combined with ultrafiltration. The results showed that the optimal extraction conditions were a liquid-to-solid ratio of 29:1 mL/g, 360 W ultrasonic treatment for 15 min, and water bath heating at 42 °C for 34 min, achieving a protein yield of 5.56%. Fan et al. [36] and Fu et al. [37] both employed ultrasound-assisted cellulase degradation to extract mulberry leaf protein. The results indicated that the optimal extraction conditions were solid-to-liquid ratio of 1:28 (g/mL), extraction solvent of 0.9% NaCl solution, 150 W ultrasound treatment for 30 min, 50 °C water bath extraction for 50 min, addition of 4.5% cellulase, enzymatic hydrolysis at 50 °C for 55 min, and acid precipitation of protein at pH 2.0. Under these parameters, the extraction yield of mulberry leaf protein could reach 16.06%. Ren et al. [38] selected mulberry leaf powder as the starting material and employed ultrasound-assisted sequential extraction using water, sodium chloride solution, ethanol, and sodium hydroxide solution as solvents to successfully obtain four types of mulberry leaf proteins: albumin, globulin, prolamin, and glutelin. The study revealed that ultrasonic treatment could enhance the extraction efficiency and antioxidant properties of albumin and globulin.

Deng et al. [39] explored the microwave-assisted extraction method of MLP and determined the optimal process conditions: pH 11.4, solid-to-liquid ratio 1:102 g/mL, microwave power 500 W, and microwave extraction time 82 s. Under these conditions, the yield of MLP reached 7.23%. Notably, this parameter combination balances extraction efficiency and protein structural stability. Their single-factor experiments revealed that microwave extraction time exceeding 80 s led to a decrease in extraction yield, which was attributed to thermal denaturation of soluble proteins—prolonged heating caused exposure of hydrophobic groups and aggregation, reducing soluble protein content. Similarly, microwave power higher than 500 W resulted in a drop in extraction yield, likely due to excessive energy input that disrupted protein molecules and co-extracted more impurities. Thus, the 500 W/82 s condition was optimized to minimize protein denaturation while maximizing extraction efficiency.

Zhao et al. [40] investigated the effects of various extraction methods (including traditional methods, ultrasonic technology, cellulase enzymatic hydrolysis, and ultrasonic-assisted cellulase enzymatic hydrolysis) on the extraction yield of MLP. Their study revealed that the strategy combining multi-frequency ultrasound with cellulase extraction demonstrated optimal efficiency. Further exploration revealed that the ultrasonic effect increased the binding frequency between cellulase and the substrate, thereby enhancing their affinity and promoting MLP dissolution. While these advanced extraction techniques offer advantages such as reduced extraction time and mild operating conditions, challenges remain regarding protein denaturation risks, equipment costs, energy consumption, and scalability for industrial production [41]. Future research should focus on balancing extraction efficiency with protein quality preservation, assessing environmental and economic impacts, and developing integrated processes that combine ultrasound/microwave technologies with enzymatic treatments for optimized, sustainable MLP extraction. Moreover, current research predominantly uses power (W) as the sole indicator of ultrasonic treatment intensity without specifying the corresponding frequency values, which poses challenges for precise experimental replication and optimization. Future studies should clearly define the frequency parameters of ultrasonic treatment and thoroughly investigate the influence mechanisms of different ultrasonic frequencies on MLP extraction.

## 4. Properties of MLP

### 4.1. The Nutritional Value of MLP

**Figure 4:**
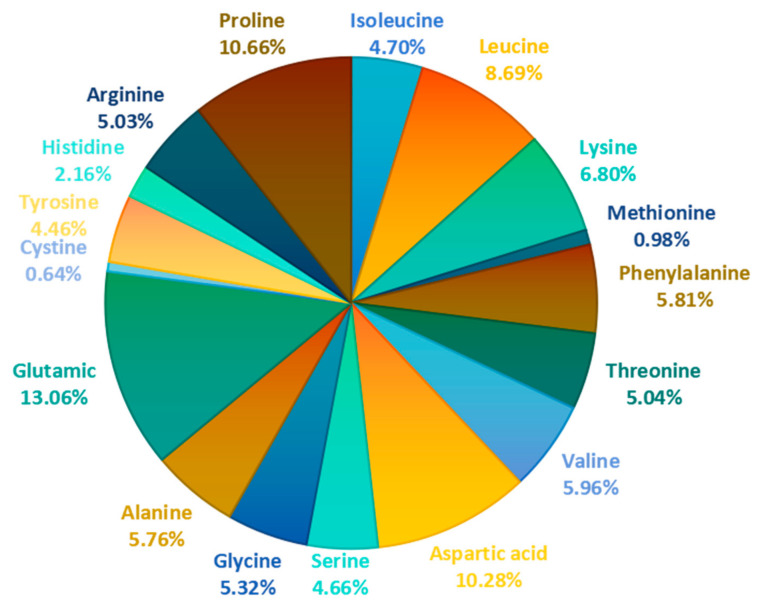
Each amino acid proportion in MLP.

The amino acid composition of MLP is presented in Table 2 and Figure 4. It is evident that MLP contains 17 different amino acids, including all seven essential amino acids required by the human body [46]. Unfortunately, tryptophan was not detected. This is likely because the indole ring structure of tryptophan is prone to hydrolysis-induced ring opening, deamination, or the formation of certain indole derivatives, making it impossible to accurately determine [47]. Currently, all the studies collected that measure the amino acid content of MLP use acid hydrolysis, which results in tryptophan remaining undetected. Future research could further refine the amino acid composition profile of MLP through alkaline hydrolysis. A balanced amino acid composition is a key factor in determining the nutritional quality of proteins [56,57]. In MLP, the proportion of essential amino acids to total amino acids (EAA/TAA) is approximately 0.38, while the ratio of essential amino acids to non-essential amino acids (EAA/NEAA) is about 0.61. Notably, these ratios are very close to the ideal protein standards established by the Food and Agriculture Organization (FAO) and the World Health Organization (WHO), which stipulate an EAA/TAA value of 0.40 and an EAA/NEAA value of 0.60 [58]. This alignment underscores the exceptional nutritional value of MLP.

Wang et al. [59] employed the amino acid ratio coefficient method to conduct a comprehensive evaluation of MLP’s nutritional value, with detailed results presented in Table 3. They calculated the amino acid ratio coefficient (RC), the ratio of amino acids (RAA), and the score of the ratio coefficient (SRC). This study identified methionine + cysteine as the primary limiting amino acids in MLP. Among its amino acid composition, the levels of leucine, isoleucine, lysine, threonine, and valine all met the standard protein levels set by FAO/WHO, while phenylalanine + tyrosine was particularly abundant. Additionally, the SRC value of MLP reached approximately 69, demonstrating its superior quality as a plant protein source and its promising development potential.

### 4.2. Functional Properties of MLP

Proteins possess certain physicochemical properties that influence food quality during processing and storage [60]. These characteristics, known as protein functional properties, are closely related to the intrinsic nature of the proteins themselves as well as the environment in which they exist.

Wang et al. [61] found that MLP exhibited excellent water-holding capacity, solubility, emulsifying properties, emulsion stability, and foaming ability under conditions deviating from the isoelectric point (i.e., at pH 5). Its water retention capacity and foaming performance showed a positive correlation with sodium chloride concentration, which ranged from 0 to 1.0 mol/L. When the NaCl concentration increased from 0 mol/L to 1 mol/L, the water-holding capacity of MLP rose from approximately 1.8 g/g to about 5.0 g/g. Meanwhile, the foaming rate increased from around 90% to approximately 120%. However, when the ionic strength was too high, specifically when the sodium chloride concentration exceeded 0.6 to 0.8 mol/L, the emulsifying function and emulsion stability were weakened. The addition of sucrose could enhance the water-holding capacity of MLP to some extent, but it reduced the solubility and foaming ability of MLP, with limited impact on emulsifying properties and emulsion stability. As the temperature increased from 4 °C to 80 °C, the oil absorption capacity and foaming ability of MLP improved, while their water-holding capacity, solubility, emulsifying properties, and emulsion stability reached optimal levels at 60 °C. These findings underscore the sensitivity of MLP functional properties to environmental factors such as pH, ionic strength, temperature, and the presence of additives, which must be carefully controlled to optimize their performance in food systems. Additionally, studies have revealed that different precipitation methods and drying techniques applied to mulberry leaves significantly affect the composition, functional characteristics, and structural features of their proteins (*p* < 0.05). This point has been previously discussed by us and will not be reiterated here [46,47,48].

With its unique hydrophilic-lipophilic properties and outstanding emulsification capabilities, MLP demonstrates the remarkable potential for applications in preparing food-grade Pickering emulsions [62]. Zhi et al. [63] prepared cross-linked MLP samples with transglutaminase (TGase) concentrations ranging from 0 to 25 U/g. Under the conditions of pH 8, a cross-linking temperature of 50 °C, a TGase concentration of 20 U/g, and an optimal cross-linking time of 60 min, an 80% *v*/*v* high internal phase Pickering emulsion was formed. This emulsion exhibited a relatively uniform droplet distribution with a unimodal size profile under such optimal crosslinking conditions, and its mean surface area diameter (d_3_,_2_) reached the minimum value, which is indicative of excellent emulsification activity. Moreover, the study demonstrated that TGase-induced cross-linking not only improved the emulsifying activity of the MLP emulsion but also enhanced its stability. Multiple lines of evidence substantiated this: the increased continuous-phase viscosity of the MLP solution (attributed to cross-linking-induced changes in surface charge and steric hindrance of the MLP adsorption layer) strengthened the system’s cohesion; the uniform droplet distribution reduced the tendency for droplet coalescence; and storage stability tests showed that the emulsion under these conditions had the lowest creaming index (CI), remaining free of obvious stratification even after 30 days of storage. These results collectively confirm the improved stability of the TGase-cross-linked Pickering emulsion.

### 4.3. Biological Activity of MLP

#### 4.3.1. Antioxidant Properties

The oxidative damage caused by free radicals is closely associated with numerous diseases, such as cancer, cardiovascular diseases, and atherosclerosis [64]. Studies by Yu et al. [65] and Zhao et al. [66] demonstrate that MLP exhibits antioxidant effects. Sun et al. [67,68] further showed that moderate enzymatic hydrolysis enhances the antioxidant activity of MLP, with the hydrolysate obtained from MLP treated with neutral protease (NH) exhibiting the highest antioxidant activity. The enzymatic hydrolysate was mainly composed of small peptides with a molecular weight of 0.3~0.6 kDa and polypeptides with a molecular weight of 0.6~6.5 kDa. Specifically, in the DPPH radical scavenging assay, NH showed an IC_50_ value of 87 µg/mL, which was lower than that of compound protease hydrolysate (PrH, 91 µg/mL) and alkaline protease hydrolysate (AH, 92 µg/mL). For superoxide anion (O_2_^−^) scavenging activity, NH also demonstrated superior performance with an IC_50_ of 351 µg/mL, compared to AH (378 µg/mL) and PrH (411 µg/mL). In the ABTS+ radical scavenging assay, NH still had the lowest IC_50_ (816 µg/mL) among the three, followed by PrH (844 µg/mL) and AH (876 µg/mL). These IC_50_ values demonstrate the stronger antioxidant capacity of neutral protease-treated MLP hydrolysate, facilitating comparison across different protein hydrolysates. They also investigated the effects of gastrointestinal digestion on MLP and its hydrolysates, finding that while gastrointestinal digestion enhances the antioxidant efficacy of MLP, it reduces the antioxidant efficiency of MLP hydrolysates [69].

The hydrolysate of MLP (HMP) effectively inhibits erythrocyte hemolysis; at a concentration of 0.4 mg/mL, the inhibition rate reaches 92% relative to the AAPH-induced hemolysis model (where AAPH alone induces severe erythrocyte hemolysis with an inhibition rate as low as 52.32%) [70]. This inhibition effect shows no significant difference (*p* > 0.05) from the normal control group (PBS-treated erythrocytes without AAPH stimulation), indicating that HMP can almost completely reverse AAPH-induced hemolytic damage at this concentration. It reduces oxidative hemolysis damage induced by 2,2′-Azobis(2-methylpropionamidine) dihydrochloride (AAPH) by suppressing malondialdehyde formation, maintaining the balance between glutathione (GSH) and oxidized glutathione (GSSH), and preserving the activity of antioxidant enzymes such as superoxide dismutase (SOD), catalase (CAT), and cellular glutathione peroxidase (GSH-Px) [71].

Similarly, Ren et al. [72] found that MLP hydrolysates could alleviate morphological abnormalities in hepatocytes damaged by H_2_O_2_, improve cell survival rates, reduce the release of lactate dehydrogenase (LDH), aspartate aminotransferase (AST), and alanine aminotransferase (ALT) in the culture medium, decrease intracellular malondialdehyde (MDA) content, and increase superoxide dismutase (SOD) activity. Resveratrol (Res) and chlorogenic acid (Cla) are endogenous phenolic compounds found in mulberry leaves. MENG et al. [73] discovered that the interaction between Res, Cla, and MLP altered the secondary structure of MLP, enhanced its thermal stability, inhibited the hydrolysis of MLP by digestive enzymes, and improved MLP’s scavenging capacity against DPPH free radicals and ABTS cation radicals. These findings collectively highlight the potent antioxidant capacity of MLP and its hydrolysates, which is mediated through multiple mechanisms, including free radical scavenging, enzyme activity modulation, and cellular protection against oxidative stress. The enhancement of antioxidant activity by enzymatic hydrolysis and gastrointestinal digestion underscores the importance of processing methods on bioactivity. However, MLP has certain antinutritional factors like phytates, tannins, or alkaloids. Even if these compounds are present in low amounts in MLP, they may still have some impact on the antioxidant properties, which requires further experimentation to demonstrate. Additionally, while in vitro and cellular studies provide promising evidence, further in vivo investigations and clinical trials are necessary to confirm the bioavailability, efficacy, and safety of MLP antioxidants in human health.

#### 4.3.2. Inhibiting ACE Activity

An important factor in hypertension is angiotensin-converting enzyme (ACE) [74]. Within the operational framework of the renin-angiotensin system (RAS), ACE plays a pivotal role by converting inactive angiotensin-I into its active form, angiotensin-II [75]. This conversion triggers vasoconstriction, leading to increased blood flow. Additionally, this condition has been shown to exacerbate the retention of salt and water in the body while reducing renal excretion of both. The net effect of these actions causes contraction in vascular smooth muscle, ultimately inducing hypertension [76,77].

Recent studies have highlighted the potential of MLP hydrolysates as natural ACE inhibitors. Chen et al. [78] conducted an in-depth study and discovered that peptides derived from the hydrolysis of MLP using flavor protease could effectively and competitively inhibit ACE activity, thus lowering blood pressure. Similarly, JIA et al. [79] employed acidic, neutral, and alkaline proteases to hydrolyze MLP and found that all enzymatic hydrolysates exhibited ACE inhibitory activity, with the hydrolysate from neutral protease showing the most significant efficacy (*p* < 0.05). Under specific process conditions—namely, a substrate concentration of 20 g/L, an enzyme dosage of 7.5%, a hydrolysis time of 50 min, a hydrolysis temperature of 55 °C, and a pH of 7.0—the ACE inhibitory activity reached as high as 81.23%, while the degree of hydrolysis achieved an impressive level of 21.41%. Taken together, mulberry leaf protein hydrolysates represent a valuable source of natural ACE inhibitory peptides, with enzymatic hydrolysis conditions and peptide molecular characteristics playing key roles in optimizing bioactivity. However, to date, research on MLP-derived peptides in this context remains limited: while in vitro assays confirm their ACE inhibitory potential, data on their in vivo stability, intestinal absorption rates, or serum bioavailability are scarce. And advancing their application as functional ingredients for hypertension management requires addressing critical knowledge gaps in their in vivo bioavailability and the translational validity of in vitro activity.

#### 4.3.3. Other Bioactivities

Diabetes is a metabolic and endocrine system disorder characterized by abnormally elevated blood glucose levels, with over 90% of all cases classified as type 2 diabetes (T2D) [80]. Feng et al. [81] found that mulberry leaf peptides obtained through MLP hydrolysis exhibit significant hypoglycemic effects (*p* < 0.05), indicating that MLP has great potential for developing healthy foods aimed at maintaining blood glucose homeostasis. Building on this foundation, Cao et al. [2] conducted a detailed investigation into the specific effects of various enzymatic hydrolysis processes on the in vitro hypoglycemic activity of mulberry leaf peptides. They designed comparative experiments encompassing products obtained from MLP hydrolyzed by complex protease, flavor protease, alkaline protease, trypsin, neutral protease, and papain, systematically evaluating their respective in vitro hypoglycemic activities. The results indicated that the product resulting from neutral protease hydrolysis displayed the most pronounced inhibition of α-glucosidase activity, with an IC_50_ of 3.52 mg/mL. This inhibition of α-glucosidase is particularly relevant as it delays carbohydrate digestion and glucose absorption, thereby contributing to postprandial blood glucose regulation. The specificity of neutral protease in generating bioactive peptides with potent enzyme inhibitory effects highlights the importance of selecting appropriate enzymatic treatments to maximize therapeutic potential [82,83]. However, current studies primarily use IC_50_ to quantify the inhibitory effect of MLP on α-glucosidase, lacking kinetic parameters that could reflect the inhibition mode and enzyme affinity.

An increase in total serum cholesterol is considered an independent risk factor for atherosclerotic cardiovascular diseases [84]. Duan et al. [85] revealed that mulberry leaf peptides obtained through alkaline protease hydrolysis exhibit significant cholesterol-lowering effects (*p* < 0.05). Subsequent studies optimized this preparation process, achieving a mulberry leaf peptide concentration of 0.262 mg/mL under optimal conditions, with cholesterol inhibition reaching as high as 33.27%. This cholesterol-lowering effect may be mediated by the ability of MLP-derived peptides to modulate lipid metabolism pathways, including inhibiting the 3-hydroxy-3-methylglutaryl coenzyme A reductase (HMGCR) and reducing the solubility of cholesterol micelles in water [86], although the precise mechanisms warrant further investigation.

Colitis is a common chronic inflammatory bowel disease with severe complications, including anemia, malnutrition, infection, and an increased risk of colon cancer [87]. Sun et al. [88] found that MLP hydrolysates can effectively alleviate symptoms of colitis. These hydrolysates significantly reduce the concentration of pro-inflammatory cytokines (*p* < 0.05), effectively repair tissue damage, and markedly increase short-chain fatty acids (SCFAs) content—specifically, acetate, n-butyrate, and branched-chain fatty acids (BCFAs) were significantly elevated compared to the DSS-induced colitis group and normal group (*p* < 0.05). Quantitatively, the MLP hydrolysate intervention (especially the preventive and therapeutic groups, PHD) increased acetate to approximately 1.6 mmol/mg, n-butyrate to approximately 1.0 mmol/mg, and total SCFAs to approximately 5.0 mmol/mg in cecal contents, with significant differences compared to group D (*p* < 0.05). Further investigation revealed that this hydrolysate demonstrates its colitis-alleviating effects through a dual regulatory mechanism involving gut microbiota and inflammatory responses. Overall, these diverse bioactivities of MLP and its hydrolysates, including hypoglycemic, cholesterol-lowering, and anti-inflammatory effects, underscore their multifaceted potential in managing metabolic and inflammatory disorders. Future research should focus on elucidating molecular mechanisms, optimizing peptide production, and validating efficacy through in vivo and clinical studies to support their development as functional food ingredients or nutraceuticals.

Additionally, MLP exhibits potent immune regulatory activity. Research by Sun et al. [89] demonstrated that MLP hydrolysate boosts the expression levels of nitric oxide (NO), interleukin-6 (IL-6), and tumor necrosis factor-alpha (TNF-α). It also markedly enhances macrophage production of reactive oxygen species (ROS) and substantially increases their neutral red pinocytic capacity. These robust changes clearly indicate the hydrolysate’s ability to activate macrophage-mediated immune responses. While paradoxically classified as pro-inflammatory cytokines, IL-6 and TNF-α play indispensable roles in launching innate immune defenses and regulating inflammatory homeostasis. The concurrent enhancement of antioxidant activity—established in previous studies—suggests a fascinating potential balance between the hydrolysate’s immunomodulatory effects and oxidative stress regulation. This delicate interplay implies that moderate induction of these key cytokines might bolster immune surveillance effectively while preventing excessive inflammation.

## 5. Summary and Prospects

As the global population continues to grow, mulberry leaf protein (MLP) emerges as a promising plant protein with significant development potential. The abundant yield of mulberry leaves, combined with their low cost, provides a solid foundation for the large-scale production of MLP. With advancements in MLP production technology, the technical support for its preparation has become increasingly robust. Furthermore, MLP is recognized for its high nutritional value, excellent functional properties, and diverse bioactivities, including antioxidant effects, blood sugar reduction, cholesterol-lowering, colitis alleviation, and ACE inhibitory activity. These characteristics greatly expand the application potential of MLP.

Despite these promising attributes, several challenges must be addressed to fully realize the commercial and therapeutic potential of MLP. Currently, most preparation techniques are limited to laboratory-scale applications, with minimal translation to industrial-scale production. Therefore, developing scalable, cost-effective, and environmentally sustainable extraction and purification processes is critical. Innovations such as enzyme-assisted extraction, ultrasound/microwave-assisted methods, and integrated bioprocessing may offer viable pathways for scaling up production.

Numerous scientific studies have confirmed that MLP and its enzymatic hydrolysates possess promising bioactive functions; however, a clear and comprehensive understanding of their mechanisms is still lacking. Additionally, regarding the potential allergenicity of MLP, particularly the issue of pathogenesis-related proteins that may be present in leaf proteins as potential allergens, this indeed represents a critical gap in current research. To enhance our knowledge of their principles of action and application methods, future research should focus on elucidating how MLP and its hydrolysates exert their bioactivities and whether mulberry leaf protein has potential allergenicity.

In particular, multi-omics approaches, including proteomics, metabolomics, and gut microbiome analysis, combined with advanced molecular docking and in vivo models, could provide deeper insights into the interactions, bioavailability, and therapeutic pathways of bioactive peptides. Such a mechanistic understanding will be essential for regulatory approval and consumer acceptance.

Currently, the variety of functional products developed from MLP is insufficient, and its application in the food industry remains in its early stages. Future research should prioritize the study and application of MLP in food products, aiming to enhance protein content while improving processing characteristics. Exploring novel food formulations, such as plant-based meat analogs, protein-enriched beverages, and functional snacks incorporating MLP, could accelerate its market penetration. Additionally, investigating the effects of MLP on sensory attributes, shelf life, and nutritional stability will be crucial for optimizing consumer acceptance.

If these challenges are overcome, MLP and its enzymatic hydrolysates will demonstrate great potential as ideal protein sources for functional foods with therapeutic benefits. Ultimately, integrating MLP into the global protein supply chain could contribute to sustainable nutrition, addressing food security and health challenges simultaneously and positioning mulberry leaf protein as a valuable resource in the future of food and medicine. This section describes the experimental results, their interpretation, as well as the experimental conclusions that can be drawn.

## Figures and Tables

**Table 1:** The different methods used in MLP extraction.

Method		MLP Yield (From Single Studies)				Key Advantages	Limitations	Reference
		Yield (%)	Dry/Wet Weight Basis	Protein Content Determination Method	Number of Repetitions			
Alkali–acid precipitation method		5.17	wet	Precipitation method	5	This method is simple and cost-effective.	The MLP obtained is prone to denaturation, resulting in a relatively low yield and purity.	[18,19,20]
Salting-out method		1.38	wet	Precipitation method	3	This method does not compromise protein bioactivity and better preserves the nutritional value of MLP.	The yield is relatively low.	[21]
Microbial fermentation		16.29	dry	Precipitation method	3	This method does not require heating and generates no waste or pollution.	The fermentation process is time-consuming and may result in nutrient loss; the production costs are higher.	[22,23]
Foam separation method		15.50	dry	Coomassie Brilliant Blue colorimetric method	5	Simple equipment, straightforward operation, low energy consumption, environmental friendliness, and high efficiency.	When tasked with separating high-concentration solutions, the efficiency of this technology appears to be relatively low.	[24,25,26,27]
Cellulase-assisted extraction method		0.98	dry	Coomassie Brilliant Blue colorimetric method	3	This extraction technique exhibits high selectivity and mild reaction conditions.	Relatively slow extraction rates and the tendency to introduce enzyme protein impurities during operation, which affects purity.	[28,29]
Ultrasound/microwave-assisted extraction method	Ultrasound-assisted salting-out extraction	9.19	dry	Coomassie Brilliant Blue colorimetric method	3	Reduced extraction time and mild operating conditions.	Protein denaturation risks, equipment costs, energy consumption, and scalability for industrial production.	[30,31,32,33,34,35,36,37,38,39,40,41]
	Ultrasound-assisted alkali dissolution and acid precipitation	5.68	dry	Coomassie Brilliant Blue colorimetric method	3			
	Ultrasonic extraction combined with ultrafiltration	5.56	dry	Coomassie Brilliant Blue colorimetric method	3			
	Ultrasound-assisted cellulase degradation	16.06	dry	Precipitation method	3			
	Microwave-assisted extraction	7.23	dry	Coomassie Brilliant Blue colorimetric method	3			

**Table 2:** The amino acid composition of MLP.

Amino Acids	Content(mg/g)	Proportion	Amino Acids	Content (mg/g)	Proportion
Isoleucine	19.3	4.70%	Glycine	21.84	5.32%
Leucine	35.68	8.69%	Alanine	23.65	5.76%
Lysine	27.92	6.80%	Glutamic	53.65	13.06%
Methionine	4.01	0.98%	Cystine	2.64	0.64%
Phenylalanine	23.85	5.81%	Tyrosine	18.34	4.46%
Threonine	20.72	5.04%	Histidine	8.86	2.16%
Valine	24.47	5.96%	Arginine	20.65	5.03%
Aspartic acid	42.22	10.28%	Proline	43.79	10.66%
Serine	19.16	4.66%	TAA ^1^	410.77	100.00%
EAA ^2^/TAA	0.38		EAA/NEAA ^3^	0.612	

^1^ TAA refers to total amino acids. ^2^ EAA includes isoleucine, leucine, lysine, methionine, phenylalanine, threonine, and valine. ^3^ NEAA includes glycine, alanine, glutamic acid, cysteine, tyrosine, histidine, arginine, aspartic acid, proline, and serine.

**Table 3:** Amino acid ratio coefficient of MLP.

Characteristic Value	FAO/WHO Essential Amino Acid Composition							SRC
	Leucine	Lysine	Phenylalanine + Tyrosine	Threonine	Valine	Isoleucine	Methionine + Cystine	
RAA	0.52	0.52	0.76	0.52	0.50	0.50	0.22	69.29
RCAA	1.03	1.02	1.49	1.03	1.00	0.98	0.44	

## Data Availability

No new data were created or analyzed in this study. Data sharing is not applicable to this article.

## Abbreviations

The following abbreviations are used in this manuscript:

MLP	mulberry leaf protein
FAO	Food and Agriculture Organization
WHO	World Health Organization
EAA	essential amino acids
TAA	total amino acids
NEAA	non-essential amino acids
RC	ratio coefficient
RAA	ratio of amino acid
SRC	score of the ratio coefficient
SRCAA	standard ratio coefficient of amino acid
ACE	angiotensin-converting enzyme
RAS	renin-angiotensin system
T2D	type 2 diabetes
DPPH	2,2-diphenyl-1-picrylhydrazyl
ABTS	2,2′-azino-bis(3-ethylbenzothiazoline-6-sulfonic acid)
TGase	transglutaminase
AAPH	2,2′-Azobis(2-methylpropionamidine) dihydrochloride
GSH	glutathione
GSSH	oxidized glutathione
SOD	superoxide dismutase
CAT	catalase
GSH-Px	cellular glutathione peroxidase
MDA	malondialdehyde
LDH	lactate dehydrogenase
AST	aspartate aminotransferase
ALT	alanine aminotransferase
Res	resveratrol
Cla	chlorogenic acid
NO	nitric oxide
IL-6	interleukin-6
TNF-α	tumor necrosis factor alpha
ROS	reactive oxygen species
NaOH	sodium hydroxide
EDTA-2Na	ethylenediaminetetraacetic acid disodium salt
CNKI	China National Knowledge Infrastructure
OpenLCA	Open Life Cycle Assessment
GBIF	Global Biodiversity Information Facility
PBS	Phosphate-Buffered Saline
PrH	compound protease hydrolysate
AH	alkaline protease hydrolysate
NH	neutral protease hydrolysate
HMP	hydrolysate of mulberry leaf protein
SCFAs	short-chain fatty acids
BCFAs	branched-chain fatty acids
PI3K	Phosphatidylinositol 3-kinase
Akt	Protein kinase B
PPARalpha	Peroxisome proliferator-activated receptor alpha
CPT-1	Carnitine palmitoyltransferase-1
IC_50_	Half maximal inhibitory concentration
